# Stress management: how does the academic staff cope with it? a cross-sectional study at the university of Udine

**DOI:** 10.1186/s12889-024-18935-7

**Published:** 2024-06-05

**Authors:** Olivia Giulia Bianca Vacchi, Diana Menis, Enrico Scarpis, Annarita Tullio, Benedetta Piciocchi, Silvia Gazzetta, Massimo Del Pin, Edoardo Ruscio, Silvio Brusaferro, Laura Brunelli

**Affiliations:** 1 Dipartimento di Area Medica, Università degli Studi di Udine, Udine, Italia; 2 Istituto di Igiene ed Epidemiologia Clinica, Azienda Sanitaria Universitaria Friuli Centrale, Udine, Italia; 3 SOC Accreditamento, Qualità e Rischio Clinico, Azienda Sanitaria Universitaria Friuli Centrale, Udine, Italia

**Keywords:** Coping, Stress management, Academia, Work, Employees, Italy

## Abstract

**Background:**

Increasing work-related stress in academia can have an impact on physical and mental health. The aim of this study was to analyse the coping strategies of staff employed at the University of Udine and to verify whether sociodemographic data, professional position, and the presence of anxiety or depression symptoms are related to the use of different coping strategies.

**Methods:**

We conducted a cross-sectional study between June and December 2020 using the Brief COPE questionnaire. We correlated coping strategies with professional position, sociodemographic data, and the presence of anxiety or depressive symptoms measured with the Patient Health Questionnaire–9 and the General Anxiety Disorder–7.

**Results:**

A total of 366 people participated in the study, including 109 junior academics, 146 senior academics, and 111 administrative staff (response rate 23.6%). The three most frequently used coping strategies in terms of approach coping style were planning (6.77 ± 1.41), active coping (6.58 ± 1.45) and acceptance (6.23 ± 1.44). Women were more likely than men to report using approach and avoidant coping strategies (*p* < 0.001). Positive reframing and religion were most commonly used by administrative staff (*p* < 0.05), in contrast to junior academics, who were more likely to use substances and self-blame (*p* < 0.05). Anxiety was found to correlate with self-blame (OR 1.94) as a coping strategy, while depression was associated with venting (OR 2.83), self-blame (OR 3.27), and humor (OR 3.02).

**Conclusion:**

Identifying profiles of coping strategies can help higher education institutions to implement support strategies for the academic community, ultimately promoting healthier lives and more effective teaching and research. Our study has shown that women and junior academics among staff at the Udine University would benefit from a tailored health promotion intervention that encourages the use of approach coping styles to reduce their risk of developing anxiety and depressive symptoms.

## Introduction

Stress is commonly described as the perception of an imbalance between the demands and the individual’s ability to respond to a situation with their resources [1]. However, stress can be divided into two different types and, according to Seyle’s definition (cited in Bienternova-Vascu et al., 2020), there is a “distress”, i.e. when the stress response is triggered by negative stressors, and a “eustress”, when the stress response is triggered by stimulating factors. In addition, the author emphasizes that stress is not what happens to someone, but how that person reacts to it [2].

When chronic stress is inadequately managed, it leads to burnout, which in turn is a risk factor for anxiety and depression [3].

The consequences of stress affect people’s health, their personal lives, and cause direct and indirect costs to the economy [4, 5], estimated by the American Psychological Association at 500 billion dollars and 550 million workdays per year in the United States [6].

Traditionally, the academic category has been characterized by low levels of stress [7], which may be related in part to the notion that autonomous/self-managed work, such as that of an academic, is less stressful because one has direct control over one’s activities, which act as a buffer against work stress [8]. However, in the last decade, academics in Italy have been given a variety of tasks related to bureaucratic and social issues as part of a national reform. The former is related to the increasing popularity of temporary contracts, management tasks [9], and financial pressures as academics are expected to attract external funding [10, 11]. Social issues include the competitive climate [5], loss of collegiality and support among colleagues [9], and lack of recognition [12].

In studies conducted at New Zealand and Australian universities, academics reported stress in up to 40% of respondents, more than general university staff [10, 12]. Some authors believe that there is a link between stress and seniority in academics [13], while others found that stress is more common in younger academics [5, 7, 14], a category that typically has job instability [15]. Considering that temporary employment is one of the main causes of stress among academics [5, 9], this issue is of great importance as PhD students, who are typically the youngest category of academics, are an important source of scientific progress [16]. The causes of stress in younger academics can also be seen in other aspects, such as lack of skills or experience in performing their own tasks and in leadership and management roles [9]. In addition, academics report the pursuit of publications [11], too much paperwork [5], inadequate salary [11], lack of promotion [5, 9], and the competing demands of career and family life [11].

Stress management interventions can be categorized as primary (i.e., to prevent stress), secondary (i.e., to reduce the severity and duration of stress), or tertiary (i.e., to rehabilitate people already suffering from a mental illness) [17]. This classification can be made at both the individual and organizational level. Some authors also refer to a third intermediate category, the individual-organizational level, which aims to change the relationship between the individual and the organization [17].

At the organizational level, interventions should focus on job redesign, which aims to change the characteristics of the workplace to improve employee well-being (e.g., workplace discretion, workload, ergonomic design) [17], or to reduce role ambiguity by creating laws and regulations that define the expectations of a particular job [18].

On an individual level, some examples are cognitive-behavioural techniques [3, 17], mindfulness [17], relaxation techniques (e.g., yoga, massage) [3, 9, 17], leisure activities [3], biofeedback [19, 20], talking to colleagues [9], and flexible working conditions (e.g., part-time work and working from home one day a week) [9, 20]. These various techniques for coping with stress at an individual level are referred to as coping strategies.

Individual ability to cope with stress also depends on personality [21], but there is a lack of systematic and comprehensive assessment of these aspects [22].

Coping strategies are indeed broad and refer to a variety of efforts to minimize the stress associated with negative life experiences (adaptive vs. maladaptive, active vs. passive, positive vs. negative, problem-oriented vs. emotion-oriented), and each of these strategies can be examined using specific questionnaires. Only a few studies have investigated coping strategies within the university [22], but most of them investigated coping strategies in a qualitative way using interviews [8, 14, 23–25], without applying an instrument with specific psychometric properties. This also makes it difficult to compare coping strategies between different studies.

As a result, there is still a lack of knowledge about the current use of coping strategies in the academic community. No data is available for the University of Udine. As this category of staff is crucial in terms of educating the new generations and for the creation of new knowledge, we can say that in universities progress and our future are being pursued and therefore studying and tracking the well-being of university staff is a priority.

Given the lack of specific quantitative data on coping with stress in the academic context, the present study therefore aimed primarily to analyse the coping strategies used by professionals in academia to deal with the main stressors they are exposed.

An accurate assessment of the coping strategies used by academic staff could be useful to implement targeted interventions to increase the resilience of the most vulnerable categories among academic staff (e.g., those who use maladaptive coping strategies).

Based on the hypothesis that age, type of employment contract, and different types of responsibilities may influence the use of specific coping strategies, we also wanted to investigate whether there was a relationship between coping style and occupational role (junior academics, senior academics and administrative staff). Referring to the literature, we wanted to confirm that age, sex, marital status, education, academic department, years of work experience, and symptoms of anxiety and depression could influence the use of different coping strategies.

This study wanted to obtain data for targeting interventions to provide prevention and support strategies for university members to increase their resilience to stress, to prevent burnout and improve psychosocial wellbeing. These data could also be useful for pursuing psychosocial well-being of academic staff in order to target interventions situated at a more organizational level.

## Method

### Study design and setting

This was a cross-sectional study conducted at the University of Udine to investigate the coping styles of academics. The study was conducted between June and December 2020. The data on coping strategies were collected as part of a cross-sectional study (UN-SAD: Symptoms of Anxiety and Depression within the UNiversity community) conducted at the University of Udine, with the aim of investigating the mental health of academics [26]. This university is attended by approximately 15,000 students annually. The university was founded in 1978 and currently (in 2023) has 692 professors and researchers, 477 technical and administrative staff. The university is located in Northeastern Italy in a region called Friuli Venezia Giulia which has about 1.000.000 inhabitants. The region borders with Austria and Slovenia and the Italian region of Veneto. The main location of the university is set in the city of Udine which counts about 98.000 inhabitants, but there are branches in Pordenone, Gemona del Friuli and Gorizia.

### Participants

All academic and administrative staff, assistants, fellows and short-term employees in all academic departments (Business and Economics, Life Sciences and Medicine, Basic Sciences and Engineering, Humanities, Political Sciences) with a total of about 1,500 eligible participants, were included in the study; only visiting professors were excluded.

### Recruitment

We sent invitations through internal academic mailing list to all institutional email addresses to participate in the survey and reminders shortly before the deadline.

### Data collection

Data collection took place between June and December 2020 via an anonymous online survey with a total of 69 items. The survey was conducted as a part of a broader study (the original UN-SAD study) [26], which aimed to identify the prevalence of depressive and anxiety symptoms among academic professionals, considering three groups: junior academics (i.e., on fixed-term contracts: researchers, post-doctoral researchers, PhD students), senior academics (i.e., on tenured contracts: full professors, associate professors, and researchers with tenured contracts), and administrative staff. It included a sociodemographic data section (age, sex, occupation, education, academic department, marital status, years of work experience and commuting distance) and two validated psychological assessment tests: Patient Health Questionnaire–9 (PHQ-9) [27] for depressive symptoms and General Anxiety Disorder–7 (GAD-7) [28] for anxiety symptoms. A special part of the survey examined coping styles using the Brief COPE (Coping Orientation to Problems Experienced) questionnaire [29]. The survey was conducted in Italian.

### Research instruments

#### Questionnaire on Coping Orientation to Problems Experienced (Brief COPE)

The Brief COPE is a widely used instrument for investigating effective and ineffective coping styles in response to stressful life events. This questionnaire has been used in different countries [24, 25, 30–37], including Italy [38], on different population groups, which is why we chose this questionnaire to ensure cross-cultural applicability and comparison with previous literature. The scale also has good psychometric properties for the Italian sample [39]. This instrument consists of 28 items divided into 14 scales. The questionnaire divides the coping strategies into denial, substance use, venting, behavioural disengagement, self-distraction, self-blame, active coping, positive reframing, planning, acceptance, seeking emotional support, seeking information support, religion, and humor. Each scale is represented by a score, which is the sum of the two items measured on a 4-point Likert scale. The scales range from two to eight: the higher the score, the more the specific coping strategy examined on the scale is used. The instrument does not offer the possibility of calculating an overall score.

The coping strategies can be divided into two large groups: the first six coping styles can be classified as avoidant coping and the second six as approach coping. Humor and religion are neither approach nor avoidant coping styles [40]. The first group includes coping styles in which the person approaches a stressor to seek information or social support, plan ahead, and try to solve the problems. The second group describes a passive strategy of moving away from a stressor, or an active strategy of moving away from or trying to escape from the stressor [40]. Specifically, six scales of the Brief COPE questionnaire address *approach coping* strategies (active coping, positive reframing, planning, acceptance, seeking emotional support, and seeking information support), while the other six scales examine *avoidant coping* strategies (denial, substance use, venting, behavioural disengagement, self-distraction, and self-blame); the two additional scales address the use of religion and humour, which are neither approach nor avoidant styles.

#### Patient Health Questionnaire–9 (PHQ-9)

This test was selected for psychological assessment because it has good sensitivity and specificity for the presence of depressive symptoms [27]. The scale has also good psychometric properties for the Italian sample [41]. The PHQ-9 is the 9-item depression module of the full PHQ. If five or more of the nine criteria for depressive symptoms were present on at least “more than half of the days” in the last two weeks and one of the symptoms is depressed mood or anhedonia, major depression can be diagnosed. If two, three, or four depressive symptoms have been present on at least “more than half of the days” in the past two weeks and one of the symptoms is depressed mood or anhedonia, other types of depression can be diagnosed. If the symptom “thoughts that you would be better off dead or thoughts about hurting yourself in some way” is present, it counts regardless of duration. Responses to the questionnaire are reported on a 3-point Likert scale, so the PHQ-9 score can range from 0 to 27, as each item can be scored from 0 (not at all) to 3 (almost every day) [27].

#### General anxiety Disorder–7 (GAD-7)

The GAD-7 is a validated test for the psychological assessment of anxiety symptoms [28]. This test was selected for psychological assessment because it has good sensitivity and specificity for the presence of anxiety symptoms. The scale has also good psychometric properties for the Italian sample [41].

The questionnaire consists of seven items asking about anxiety related problems in the last two weeks. The answers are given on a 4-point Likert scale, so that the questionnaire can range from 0 to 21 points. It serves as a screening tool for anxiety symptoms, so scores of five, 10, and 15 are considered to be the cut-off points for mild, moderate and severe anxiety, respectively. If the score is 10 or higher, further testing is recommended [28].

### Sample size

The sample size was calculated for the original UN-SAD study with a 95% confidence level and based on the hypothesis of a different prevalence of minor psychiatric disorders in the three groups (junior academics, senior academics and administrative staff) [26]. The same individuals were interviewed using the Brief COPE questionnaire, which was also included in the original UN-SAD questionnaire.

We could find no previous data on the coping strategies used by academics at the University of Udine, so we hypotized that avoidant and approach coping styles might be evenly distributed in this population. Assuming that the prevalence of approach and avoidant coping styles was 50% in each group, and using the same 95% confidence level, we calculated an accuracy of 9% with the collected UN-SAD questionnaires. The study was approved by the Institutional Review Broad of the University of Udine, Italy.

### Data analysis

Descriptive analyses were carried out for all variables. Data were presented as frequencies and percentages for categorical variables and as means ± standard deviations or median ± interquartile range (IQR) and minimum and maximum values for continuous variables. Results were presented as both categorical (values from two to eight) and dichotomous variables (avoidant or approach coping style). Chi-square tests and Fisher’s Exact tests were used where appropriate to assess the possible association between categorised variables. Student’s t-test, Wilcoxon–Mann–Whitney test, or Kruskal-Wallis test were used to compare continuous variables based on the Kolmogorov–Smirnov normality test. Binary univariate and multivariate logistic regression analyses were used to assess the association with dichotomous outcomes. Regression results were expressed as raw and adjusted ORs with 95% CI and *p*-values. The significance level was set at 0.05. All statistical analyses were performed using R. software, version 3.4.2 (R Foundation for Statistical Computing, Vienna, Austria) [R: The R Project for Statistical Computing. Available at: https://www.r-project.org/. [Last accessed 2022 Aug 13]].

## Results

A total of 366 academic professionals responded to our survey, which corresponds to a response rate of 23.6% (366/1,550). The professional groups were evenly distributed between senior academics (N. 145; 39.9%), administrative staff (N. 111; 30.3%), and junior academics (N. 109; 29.8%). The majority of respondents in the groups of junior academics and administrative staff were women, namely 53.2% (N. 58) and 77.5% (N. 86), respectively, while the senior academics were predominantly men (N. 86; 58.9%). The mean age of the participants was 47.9 ± 12.0 years, with the junior academics being younger than the others (33.2 ± 6.4 years). The majority of respondents among senior academics and administrative staff were married/cohabiting (79.5% and 78.0%, respectively), while most junior academics (61.5%) were single. Thirty-three (9%) of respondents were divorced/separated or widowed. The most common position among senior academics was associate professor (N. 70; 47.9%), while junior academics were mostly fellows (N. 55; 50.5%). The sociodemographic characteristics of the respondents are presented in Table 1. The full description of the characteristics of the respondents is included in the original article by Scarpis et al. [26].

**Table 1:** Sociodemographic characteristics of respondents

Sociodemographic characteristics	Senior Academics	Junior Academics	Administrative staff	Overall
	(*N* = 146)	(*N* = 109)	(*N* = 111)	(*N* = 366)
**sex n (%)**				
Female	60 (41.1%)	58 (53.2%)	86 (77.5%)	204 (55.7%)
Male	86 (58.9%)	51 (46.8%)	25 (22.5%)	162 (44.3%)
**Age (yr), mean ± SD**				
Mean ± SD	55.6 ± 7.03	33.2 ± 6.42	52.3 ± 7.69	47.9 ± 12.0
**Marital status, n (%)**				
Single	15 (10.3%)	67 (61.5%)	16 (14.4%)	98 (26.8%)
Divorced/separated	13 (8.9%)	1 (0.9%)	13 (11.7%)	27 (7.4%)
Married/cohabiting couples	116 (79.5%)	41 (37.6%)	78 (70.3%)	235 (64.2%)
Widowed	2 (1.4%)	0 (0%)	4 (3.6%)	6 (1.6%)
**Educational level, n (%)**				
PhD	105 (71.9%)	69 (63.3%)	6 (5.4%)	180 (49.2%)
Medical specialty	3 (2.1%)	1 (0.9%)	0 (0%)	4 (1.1%)
Doctor or equivalent	3 (2.1%)	0 (0%)	6 (5.4%)	9 (2.5%)
Master’s or equivalent	35 (24.0%)	39 (35.8%)	54 (48.6%)	128 (35.0%)
Bachelor’s or equivalent	0 (0%)	0 (0%)	7 (6.3%)	7 (1.9%)
Upper secondary education	0 (0%)	0 (0%)	38 (34.2%)	38 (10.4%)
**Profile, n (%)**				
Associate professor	70 (47.9%)	/	/	70 (19.1%)
Full professor	39 (26.7%)	/	/	39 (10.7%)
Senior researcher	37 (25.3%)	/	/	37 (10.1%)
Fellow	/	55 (50.5%)	/	55 (15.0%)
PhD student	/	32 (29.4%)	/	32 (8.7%)
Junior researcher	/	22 (20.2%)	/	22 (6.0%)
**Department, n (%)**				
Business and Economics	22 (15.1)	22 (15.1)	/	30 (8.2)
Life Sciences and Medicine	14 (9.6)	14 (9.6)	/	29 (7.9)
Basic Sciences and Engineering	84 (57.5)	84 (57.5)	/	150 (41.0)
Humanities	23 (15.8)	23 (15.8)	/	41 (11.2)
Political Sciences	2 (1.4)	2 (1.4)	/	3 (0.8)
Missing	1 (0.7)	1 (0.7)	/	111 (30.3)
**Years of working experience (yr), mean ± SD**				
Mean (SD)	25.0 ± 8.1	5.72 ± 5.10	24.7 ± 8.87	19.2 ± 11.6

As shown in Table 2, the three most frequently used coping strategies in terms of approach coping style were planning (mean ± SD, 6.77 ± 1.41), active coping (6.58 ± 1.45), and acceptance (6.23 ± 1.44). The least used strategies related to avoidant coping styles included behavioural disengagement (2.90 ± 1.19), denial (2.49 ± 0.93), and substance use (2.20 ± 0.78). Overall, all other coping styles belonging to the approach and avoidant groups were used about equally often. Humour and religion were also in the middle range, although they were among the three least common. Women were more likely than men to use approach coping strategies based on external support - informational support (5.31 ± 1.54) and emotional support (5.09 ± 1.57), positive reframing (5.51 ± 1.63), and planning (6.88 ± 1.40), and avoidant coping styles such as self-distraction (5.28 ± 1.57), venting (5.10 ± 1.53), and self-blame (5.88 ± 1.38). Women were also more inclined to use religion than men (3.64 ± 1.91). In terms of occupational groups, positive reframing (5.59 ± 1.73) and religion (4.05 ± 2.03) were most frequently used by administrative staff compared to junior and senior academics. In contrast, junior academics were less likely than others to use acceptance (5.89 ± 1.43) and planning (6.48 ± 1.44), while they were more likely to use substances (2.46 ± 1.24) and self-blame (5.92 ± 1.58). Finally, senior academics were found to be less likely to use the denial strategy (2.35 ± 0.78) than younger colleagues and administrative staff.

**Table 2:** Brief COPE results for the total sample, divided by gender and occupational group

BRIEF COPE - INVESTIGATED COPING STRATEGY		Sex			Occupational group			
Scale	**Overall**	**Women**	**Men**	*p*-value*	**Senior academics**	**Junior academics**	**Administrative** **staff**	*p*-value**
	**(N = 366)**	**(N = 204)**	**(N = 162)**		**(N = 146)**	**(N = 109)**	**(N = 111)**	
	Mean (SD)	Mean (SD)	Mean (SD)		Mean (SD)	Mean (SD)	Mean (SD)	
	Median (IQR) [Min, Max]	Median (IQR) [Min, Max]	Median (IQR) [Min, Max]		Median (IQR) [Min, Max]	Median (IQR) [Min, Max]	Median (IQR) [Min, Max]	
**APPROACH COPING STYLE**								
Positive reframing	5.19 (1.69)	5.51 (1.63)	4.78 (1.69)	**< 0.001**	5.15 (1.64)	4.83 (1.65)	5.59 (1.73)	**< 0.05**
	5.00 (2.00) [2.00, 8.00]	6.00 (3.00) [2.00, 8.00]	5.00 (2.00) [2.00, 8.00]		5.00 (2.00) [2.00, 8.00]	5.00 (2.00) [2.00, 8.00]	6.00 (3.00) [2.00, 8.00]	
Use of informational support	4.97 (1.55)	5.31 (1.54)	4.55 (1.47)	**< 0.001**	4.86 (1.49)	5.11 (1.74)	4.98 (1.44)	0.48
	5.00 (2.00) [2.00, 8.00]	5.00 (2.00) [2.00, 8.00]	4.00 (2.00) [2.00, 8.00]		5.00 (2.00) [2.00, 8.00]	5.00 (2.00) [2.00, 8.00]	5.00 (2.00) [2.00, 8.00]	
Active coping	6.58 (1.45)	6.71 (1.39)	6.43 (1.50)	0.08	6.64 (1.37)	6.35 (1.51)	6.73 (1.46)	0.09
	7.00 (2.00) [2.00, 8.00]	7.00 (2.00) [2.00, 8.00]	7.00 (2.00) [2.00, 8.00]		7.00 (2.00) [3.00, 8.00]	6.00 (2.00) [2.00, 8.00]	7.00 (2.00) [3.00, 8.00]	
Emotional support	4.60 (1.69)	5.09 (1.57)	3.99 (1.64)	**< 0.001**	4.37 (1.63)	4.83 (1.80)	4.69 (1.63)	0.08
	4.00 (2.00) [2.00, 8.00]	5.00 (2.00) [2.00, 8.00]	4.00 (3.00) [2.00, 8.00]		4.00 (3.00) [2.00, 8.00]	5.00 (2.00) [2.00, 8.00]	5.00 (2.00) [2.00, 8.00]	
Acceptance	6.23 (1.44)	6.21 (1.41)	6.25 (1.48)	0.63	6.38 (1.42)	5.89 (1.43)	6.35 (1.44)	**< 0.05**
	6.00 (2.00) [2.00, 8.00]	6.00 (2.00) [2.00, 8.00]	6.00 (2.00) [2.00, 8.00]		7.00 (1.75) [2.00, 8.00]	6.00 (2.00) [3.00, 8.00]	6.00 (3.00) [2.00, 8.00]	
Planning	6.77 (1.41)	6.88 (1.40)	6.62 (1.41)	**< 0.05**	6.86 (1.35)	6.48 (1.44)	6.92 (1.43)	**< 0.05**
	7.00 (2.00) [2.00, 8.00]	8.00 (2.00) [2.00, 8.00]	7.00 (2.00) [3.00, 8.00]		7.00 (2.00) [3.00, 8.00]	7.00 (2.00) [2.00, 8.00]	8.00 (2.00) [3.00, 8.00]	
**AVOIDANT COPING STYLE**								
Self-distraction	4.98 (1.63)	5.28 (1.57)	4.61 (1.62)	**<0.001**	4.88 (1.56)	5.13 (1.55)	4.97 (1.79)	0.45
	5.00 (2.00) [2.00, 8.00]	5.00 (3.00) [2.00, 8.00]	5.00 (3.00) [2.00, 8.00]		5.00 (2.00) [2.00, 8.00]	5.00 (2.00) [2.00, 8.00]	5.00 (3.00) [2.00, 8.00]	
Venting	4.66 (1.61)	5.10 (1.53)	4.11 (1.54)	**<0.001**	4.50 (1.64)	4.60 (1.58)	4.95 (1.58)	0.10
	5.00 (2.00) [2.00, 8.00]	5.00 (2.00) [2.00, 8.00]	4.00 (2.00) [2.00, 8.00]		4.00 (3.00) [2.00, 8.00]	5.00 (3.00) [2.00, 8.00]	5.00 (2.00) [2.00, 8.00]	
Denial	2.49 (0.93)	2.52 (0.92)	2.44 (0.95)	0.22	2.35 (0.78)	2.50 (0.87)	2.67 (1.14)	**< 0.05**
	2.00 (1.00) [2.00, 8.00]	2.00 (1.00) [2.00, 6.00]	2.00 (0) [2.00, 8.00]		2.00 (0) [2.00, 6.00]	2.00 (1.00) [2.00, 5.00]	2.00 (1.00) [2.00, 8.00]	
Behavioral disengagement	2.90 (1.19)	2.91 (1.18)	2.90 (1.21)	0.93	2.84 (1.11)	3.05 (1.33)	2.85 (1.15)	0.50
	2.00 (2.00) [2.00, 8.00]	2.00 (2.00) [2.00, 7.00]	2.00 (1.00) [2.00, 8.00]		2.00 (2.00) [2.00, 6.00]	3.00 (2.00) [2.00, 8.00]	2.00 (1.00) [2.00, 8.00]	
Substance use	2.20 (0.78)	2.17 (0.69)	2.23 (0.87)	0.49	2.08 (0.37)	2.46 (1.24)	2.11 (0.45)	**< 0.05**
	2.00 (0) [2.00, 8.00]	2.00 (0) [2.00, 8.00]	2.00 (0) [2.00, 8.00]		2.00 (0) [2.00, 5.00]	2.00 (0) [2.00, 8.00]	2.00 (0) [2.00, 5.00]	
Self-blame	5.64 (1.48)	5.88 (1.38)	5.34 (1.55)	**< 0.001**	5.52 (1.45)	5.92 (1.58)	5.52 (1.39)	**< 0.05**
	6.00 (2.00) [2.00, 8.00]	6.00 (2.00) [2.00, 8.00]	5.00 (2.00) [2.00, 8.00]		5.00 (1.00) [2.00, 8.00]	6.00 (2.00) [2.00, 8.00]	5.00 (1.00) [2.00, 8.00]	
**NEITHER APPROACH NORE AVOIDANT COPING STYLE**								
Religion	3.44 (1.84)	3.64 (1.91)	3.19 (1.74)	**<0.05**	3.15 (1.71)	3.20 (1.69)	4.05 (2.03)	**< 0.001**
	2.00 (3.00) [2.00, 8.00]	3.00 (3.00) [2.00, 8.00]	2.00 (2.00) [2.00, 8.00]		2.00 (2.00) [2.00, 8.00]	2.00 (2.00) [2.00, 8.00]	4.00 (4.00) [2.00, 8.00]	
Humor	3.88 (1.47)	3.89 (1.39)	3.86 (1.56)	0.62	3.80 (1.39)	3.94 (1.48)	3.91 (1.55)	0.72
	4.00 (2.00) [2.00, 8.00]	4.00 (2.00) [2.00, 8.00]	4.00 (2.00) [2.00, 8.00]		4.00 (2.00) [2.00, 8.00]	4.00 (2.00) [2.00, 8.00]	4.00 (2.00) [2.00, 8.00]	

*Wilcoxon Rank Sum test ** Kruskal Wallis test

Multivariate analysis revealed, in a statistically significant manner, that men were less likely than women to use positive reframing (OR 0.47), information support (OR 0.34), emotional support (OR 0.3), self-distraction (OR 0.53) and self-blame (OR 0.33). The presence of anxiety, as measured by the GAD-7, was statistically significantly negatively correlated with the use of positive reframing (OR 0.45) and positively correlated with self-blame (OR 1.94) as a coping strategy. The presence of depression assessed with the PHQ-9 was statistically significantly negatively associated with the use of active coping (OR 0.32) and planning (OR 0.46), whereas there was a positive correlation with the use of venting (OR 2.83), self-blame (OR 3.27), and humor (OR 3.02). The results of the multivariate analysis are shown in Table 3. No significant association was found for age, occupation, education, academic department, years of work experience, and commuting distance. The only significant association was found with marital status: married individuals used humor less often than unmarried individuals, separated/divorced individuals used acceptance less often than unmarried individuals, and married and separated/divorced individuals used positive reframing less often than unmarried individuals.

**Table 3:** Multivariate analysis results for coping strategies, in relation to sex, anxiety (GAD-7) and depression (PHQ-9).

Brief COPE investigated Coping strategy	Sex			Anxiety (GAD-7)			Depression (PHQ-9)		
Scale	Crude OR (95%CI)	Adjusted OR (95%CI)*	*p*-value	Crude OR (95%CI)	Adjusted OR (95%CI)	*p*-value	Crude OR (95%CI)	Adjusted OR (95%CI)	*p*-value
**APPROACH COPING STYLE**									
Positive reframing	0.49 (0.32,0.76)	**0.47 (0.29,0.75)**	**0.002**	0.42 (0.24,0.73)	**0.45 (0.23,0.9)**	**0.02**	0.51 (0.31,0.85)	0.79 (0.41,1.51)	0.47
Use of informational support	0.41 (0.26,0.63)	**0.34 (0.21,0.55)**	**< 0.001**	0.9 (0.54,1.5)	0.73 (0.37,1.41)	0.34	0.96 (0.59,1.56)	0.98 (0.52,1.87)	0.96
Active coping	0.96 (0.6,1.56)	0.89 (0.53,1.5)	0.66	0.5 (0.29,0.85)	0.99 (0.49,1.98)	0.97	0.34 (0.2,0.57)	**0.32 (0.16,0.63)**	**< 0.001**
Emotional support	0.31 (0.19,0.5)	**0.3** **(0.18,0.51)**	**< 0.001**	1.34 (0.8,2.25)	0.98 (0.5,1.94)	0.96	1.36 (0.83,2.23)	1.14 (0.59,2.22)	0.69
Acceptance	1.04 (0.68,1.60)	1.0 (0.63,1.59)	0.99	0.46 (0.28,0.76)	0.61 (0.32,1.15)	0.12	0.49 (0.3,0.79)	0.73 (0.39,1.37)	0.32
Planning	0.95 (0.57,1.6)	0.83 (0.47,1.44)	0.5	0.56 (0.32,0.99)	0.91 (0.43,1.91)	0.79	0.43 (0.25,0.75)	**0.46 (0.22,0.94)**	**0.032**
**AVOIDANT COPING STYLE**									
Self-distraction	0.52 (0.33,0.83)	**0.53 (0.32,0.86)**	**0.011**	1.62 (0.97,2.71)	1.21 (0.62,2.35)	0.57	1.63 (1,2.67)	1.37 (0.72,2.62)	0.34
Venting	0.32 (0.19,0.55)	**0.33 (0.19,0.58)**	**< 0.001**	1.74 (1.02,2.97)	0.79 (0.38,1.61)	0.51	2.56 (1.54,4.26)	**2.83 (1.43,5.63)**	**0.003**
Denial	NA	NA	NA	NA	NA	NA	2.91 (0.18,47.05)	22.26 (0.61,813.46)	0.091
Behavioral disengagement	0.83 (0.23,3.01)	1.00 (0.23,4.29)	0.99	8.6 (2.17,34.04)	1.08 (0.24,4.83)	0.92	NA	NA	NA
Substance use	1.91 (0.31,11.54)	1.73 (0.25,12.14)	0.58	5.27 (0.87,32.07)	1.25 (0.14,11.56)	0.84	12.04 (1.33,109.16)	5.13 (0.37,70.75)	0.22
Self-blame	0.61 (0.4,0.94)	**0.58 (0.36,0.93)**	**0.025**	4.01 (2.39,6.74)	**1.94 (1.02,3.71)**	**0.045**	4.47 (2.71,7.38)	**3.27 (1.74,6.18)**	**< 0.001**
**NEITHER APPROACH NORE AVOIDANT COPING STYLE**									
Religion	0.63 (0.35,1.12)	0.79 (0.43,1.47)	0.46	0.83 (0.42,1.64)	0.7 (0.29,1.67)	0.41	1.06 (0.57,1.99)	1.23 (0.55,2.77)	0.61
Humor	0.84 (0.42,1.69)	0.87 (0.42,1.83)	0.72	1.3 (0.6,2.81)	0.7 (0.26,1.83)	0.46	2.16 (1.07,4.36)	**3.02 (1.24,7.4)**	**0.015**

*All binary logistic regression models have included sex, age, GAD-7, PHQ-9, profile as independent variables. NA = Not applicable, due to the low number of observations (< 3%); in bold: statistically significant values

## Discussion

The aim of our study was to investigate the use of different coping strategies to deal with stress in academia and to examine whether there is an association with professional position, sociodemographic data, and the presence of anxiety/depressive symptoms, as reported in the original paper [26].

The distribution of the study participants’ characteristics was close to the available data on Italian administrative staff, junior and senior academics [42].

Our results showed that the three most frequently used coping strategies in terms of approach coping style included planning, active coping and acceptance. The least used strategies related to avoidant coping styles included behavioural disengagement, denial, and substance use. Overall, all other coping styles belonging to the approach and avoidant groups were about equally used.

Humour and religion were also in the middle range. Similarly, adaptive coping strategies (acceptance, active coping, positive reframing) were most frequently used in the Lee et al.’s study involving students and university employees (i.e., academics and administrative) [8]. This study differs from our study in that it included both staff and students as participants. However, in a subgroup analysis, the only two coping strategies that were used more frequently among academic staff than students were positive reframing and religion. On the other hand, the study has similarities in that it was conducted during the COVID-19 pandemic. This is an important factor when considering that the use of a particular coping style is influenced by both the predisposition of the individual and the context in which it is used (e.g., social context, duration, and exposure to the stressor).

However, in a study conducted among academics at universities in Malaysia, maladaptive coping strategies were more prevalent than adaptive ones [43]. The author’s interpretation was that the stress load was so high that the assumption of an adaptive coping pattern did not work. In another recent study, the two most prevalent coping strategies among academic staff at a Northern Irish university were substance use and behavioural disengagement (both of which are avoidant strategies) [34].

However, it should be noted that these data [8, 34, 43] should be interpreted with caution as the Brief COPE questionnaire was used in different ways: some authors calculated the total score for each participant [8], others used a shortened version [34], and still others used a modified Likert scale [43].

Differences in the coping strategy used can affect people’s ability to manage problems in an effective way, leading to different individual and collective outcomes. For example, people who use active coping strategies have been found to have higher self-esteem and attempt to purposefully cope with problems by seeking social support, whereas those with passive coping strategies have lower self-esteem and seek self-imposed social isolation [37, 44]. In addition, the problem-oriented coper with active coping and planning aims to eliminate the stressor, whereas the emotion-oriented coper with venting, positive reappraisal, rumination, and self-blame aims to change their emotional response to the stressor [45].

### Gender and coping styles

Overall, we found in this study that women reported coping strategies more frequently than men. This was particularly true for some *approach coping* styles (i.e. positive reframing, use of informational support, emotional support, and planning), as well as for *avoidant coping* styles (i.e. self-distraction, venting, and self-blame). Women were also more likely to use religion to cope with stress.

In our study, we found that women were more likely than men to use external support-based approach coping strategies (i.e., informational support, and emotional support, positive reframing, and planning), and avoidant coping styles (i.e., self-distraction, venting, and self-blame), and that, they generally tended to use more coping styles than men.

Conversely, Darabi et al. found no statistically significant differences in coping strategies between men and women in British academics [30].

Likewise to our findings, Kataoka et al.’s study of gender differences found that women employed at a Japanese university were significantly more likely to use self-distraction, emotional support, informational support, behavioural disengagement, venting, and self-blame [25].

Similarly, we reported that women were generally more likely to use coping strategies than men, not only those they mentioned, but also positive reframing and planning. In contrast, we found no gender differences in behavioural disengagement.

The cluster analysis conducted by Doron et al. revealed that individuals typically fall into four subgroups [36]. The first group includes individuals who frequently seek external support and distraction (high-copers); the second group consists of participants characterized by high use of problem solving and moderate cognitive restructuring (adaptive copers); the third group is represented by individuals with high avoidance (avoidant copers); and the fourth group includes individuals with high cognitive restructuring (low-copers). Their results seem to confirm the existing difference between men and women in coping styles, as women were overrepresented in the high copers and avoidant copers groups, while men were mainly represented in the low copers group [36].

Zehra et al. investigated the coping strategies of residents in an emergency department in Pakistan [31]. Similar to our results, they found that all coping strategies, except for substance use, were predominantly chosen by women.

Our observation that women are more likely than men to use coping strategies related to emotional support confirms what Marinaki et al. found at Greek universities [46]. The study by Marinaki et al. included academic staff at Greek public universities and found that female academics were more likely to seek social support than their male counterparts. However, they did not use the same instrument to assess coping styles, which makes it difficult to compare their results with ours.

The greater use of some coping strategies by women may be related to the greater burden of mental health problems [47] and stress [37] reported to affect them.

Another reason for stress in female academics could be job role: a recent study showed that female employees at a university in gender-incongruent roles reported higher levels of stress than men in a gender-incongruent role [23].

As mentioned earlier, these differences between men and women could be due to differences in exposure to stress triggers, but also to differences in perception or reporting. Although it could be argued that women’s stress levels may be higher, the observed tendency to rely more on emotions could mean that they are more sensitive to external or internal stressors that have been reported to affect academics, such as excessive workload, job insecurity, and lack of support [22]. In addition, the fact that they seek external support to a high degree could be the reason for the increased reporting of this phenomenon. Our study did not focus on issues of equality, diversity and inclusion (EDI issues), we did not investigate whether participants felt part of a marginalized group. Unfortunately, data collected by the Equal Opportunities Committee of the University of Udine, published in their annual report [48], still confirms an important gender gap in the roles of professors and researchers (e.g., only 25% of full professors are women, and there is also a glass ceiling, as women are mainly represented at the base of the pyramid as students, and become fewer and fewer towards the top as full professors and the management levels). Consequently, it is possible that some of the stress faced by female academic staff is related to the gender gap, but since we did not investigate feelings of the marginalization, we could not relate coping style to EDI issues.

### Occupational role and coping styles

In terms of occupational groups, positive reframing and religion were most frequently used by administrative staff compared to junior and senior academics, while junior academics resorted to substances and self-blame more frequently than others. Finally, senior academics were found to use the denial strategy less frequently than junior colleagues and administrative staff.

In contrast to the reports of Marinaki et al. [46], we found different patterns of coping styles among administrative staff, junior and senior academics. To some extent, the differences between junior and senior academics may also be considered as differences in experience, as senior academic positions are often given to more experienced professionals. Some studies suggest that coping strategies may change over the course of a career, shifting from a problem-oriented coping strategy to an emotion-oriented coping strategy [49, 50]. This could be due to different problems that a person faces at different stages of their career, e.g., decisions in the early career years that may be crucial for the development of the young academic, while activities in the late career years are more routine. Some authors argue that this difference is related to age, as younger people experience life-changing events (e.g., marriage, birth of children), whereas older people mostly experience routine or loss events (e.g., loss of health or loved ones) [49]. Another study conducted in an academic setting that indirectly examined the differences between the coping strategies of different age groups is that of Lee et al. In their case, the older group of administrative and academic staff used positive reframing and religion more frequently than the younger group of students [8]. Similarly, we showed that positive reframing and religion were most frequently used by administrative staff compared to junior and senior academics.

In addition, our study showed that junior academics were less likely than others to use acceptance and planning, whereas they were more likely to use substances and self-blame. A higher prevalence of stress [5] and depression [26] has already been reported in younger academics. In addition, a worrying overlap has been found between burnout, depression, and substance abuse [51] and between feelings of inadequacy, hopelessness, and self-blame associated with depressed mood [52].

To cite the Lee et al. article again, we must consider that the context of the COVID-19 pandemic may have influenced the results of the study. Positions associated with a stable job that provided income during lockdown may have led to less frequent use of avoidant coping strategies such as substance use and self-blame compared to more unstable positions, such as those held by junior academics [8].

### Mental health and coping styles

Our findings suggest that there is some association between mental health status (anxious or depressed) and the coping strategies that individuals typically use, as was also the case in the study by Kataoka et al. [24]. In both studies, an association was found between mental health problems and the use of avoidant coping styles, although not in relation to the same strategies, with the exception of self-blame. As in our study, Kataoka et al. also found a significant correlation between anxiety and self-blame [24]. Although coping styles were measured with the same instrument, the limitation of this comparison is that anxiety symptoms were assessed with different questionnaires.

On the other hand, our results differ from those previously reported in the study by Batsikoura et al. [35]. In their case, the use of denial, behavioural disengagement, and substances were positively correlated with anxiety scores, whereas humor, acceptance, and planning were negatively associated with anxiety. In the Greek general population over the age of 18, the use of self-blame was positively correlated with anxiety, similar to our results, and the risk of anxiety when using positive reframing was lower in the same group. However, as the questionnaire used to assess anxiety in the study by Batzikoura et al. differs from the questionnaire used in our study, a true comparison is not possible [35].

Other studies in Italian and Australian populations, confirmed that approach coping styles were associated with lower levels of anxiety and depressive symptoms, and added that avoidant coping strategies were significantly associated with higher levels of anxiety and depression [38, 53].

Also, in the study by Agha et al. in which the 28 items of the Brief COPE were categorized into four subscales (i.e., active avoidance, problem-focused, positive coping, religious/denial), there is a significant association between anxiety and depression and the subscales with avoidant coping strategies: active avoidance and religious/denial [54].

It is important to consider the social and cultural context as it may lead to relevant differences in the results. For example, in Muslim cultures, the two most commonly used coping styles were religion and acceptance, both in the general population [55] and among university students [56] and in a population with anxiety and depressive symptoms [33].

### Limitations and strengths of the study

Although the use of a validated instrument is a methodological strength, it made it difficult to compare our results with other studies conducted in academia using other instruments. In addition, we found some heterogeneity in the use of terms and classifications to describe the different coping styles (e.g., approach/avoidant, active/passive, positive/negative, adaptive/maladaptive, problem/emotion-oriented), which made such comparisons difficult. The design of our study, which was cross-sectional and involved only one academic centre, certainly represents a limitation, as the representativeness of our results may have been influenced by this choice. Furthermore, as participation in the study was voluntary, it may have been influenced by some selection bias. Nonetheless, the sample size was adequate and the results were representative of our academic community, including senior and junior academics as well as administrative staff.

Another critical aspect is that our survey did not investigate whether participants felt they belonged to a marginalized group, so we cannot assess whether academic staff belonging marginalized groups (i.e. women, ethnic minorities, disabled people, etc.) have different coping styles from the rest of the academic staff. In addition, when interpreting the results, we must bear in mind that the data was collected during the second wave of the COVID-19 pandemic. Given the reported increase in anxiety and depression during the pandemic, both nationally [57] and internationally [58], this may have influenced our results. Finally, contextual considerations regarding differences in cultural coping with stress across countries need to be made before our findings can be generalized.

## Conclusions

Coping styles remain largely unexplored in academia, but further studies such as the present one would facilitate the identification of links with risk or protective factors so that higher education institutions could be informed about what can be done to support their community. Such support is necessary to improve both the personal and professional lives of academic staff, ultimately leading to healthier lives and more effective teaching and research. Our study showed that the use of the coping style self-blame was positively related to anxiety symptoms and the use of venting and self-blame was positively related to depression symptoms. In our population, women used self-distraction, venting, and self-blame more frequently than men, whereas junior academics used substance use and self-blame more frequently than senior academics and administrative staff. This suggests that women and junior academics would benefit from a tailored health promotion and prevention intervention to encourage these populations to use more approach coping styles such as active coping and planning. Examining the complexity of coping can help identify individuals at increased risk for stress and unhealthy behaviours and develop health promotion and prevention interventions that enable people to use the most effective coping methods. Such interventions should be implemented at the organizational level with laws and regulations to improve working conditions, at the organizational-individual level with strategies such as peer support groups, and at the individual level with cognitive behavioural techniques.

## Data Availability

The datasets used and/or analyzed during the current study are available from the corresponding author on reasonable request.
